# Assessing the Efficiency of Open-System Densification on Chemically Treated *Dendrocalamus asper* Bamboo

**DOI:** 10.3390/ma18122719

**Published:** 2025-06-10

**Authors:** André Luiz Pereira de Godoy Junior, Marzieh Kadivar, Leo Maia do Amaral, Adriano Galvão de Souza Azevedo, Juan Camilo Adrada Molano, Esmaeil Biazar, Holmer Savastano Junior

**Affiliations:** 1 Research Center on Materials for Biosystems (BioSMat), Materials Science and Engineering Graduate Program, University of São Paulo, Pirassununga 13635-900, SP, Brazil; kadivar.ma@usp.br (M.K.); leoamaral@usp.br (L.M.d.A.); adrianogalvao@usp.br (A.G.d.S.A.); camiloadrada@usp.br (J.C.A.M.); holmersj@usp.br (H.S.J.); 2 BAMbuild Company, Pirassununga 13634-204, SP, Brazil; 3 Department of Biomaterials Engineering, To.C., Islamic Azad University, Tonekabon 4684161167, Iran; kianoushbiazar@gmail.com

**Keywords:** bamboo, delignification, densification, process optimization

## Abstract

The natural variability and moisture sensitivity of bamboo limit its widespread use in construction applications. To address these challenges, densification and delignification processes have emerged as promising modification techniques. Densification and delignification processes can lead to significant improvements in the physical, mechanical, and chemical properties of solid wood. In this study, a two-step process of delignification and densification was carried out on *Dendrocalamus asper* bamboo specimens. The objective was to assess whether the optimized parameters of densification for natural bamboo on an open pressing system can be transferred for delignified bamboo. Delignification was achieved using an alkali solution (NaOH and Na_2_SO_3_) with two different temperature settings (25 °C or 100 °C). The pre-treated samples were dried in one of the two different conditions, either at 100 °C for 24 h or 25 °C for 30 days, resulting in four different groups with an average moisture content ranging from 7 to 10%. The samples were densified to 50% of their original thickness through an open thermo-mechanical press system at 160 °C with a compression rate of 6.7 mm/min and compared to densified bamboo without delignification (reference). The compression stress required to achieve a 50% degree of densification was evaluated, with untreated samples exhibiting an average value close to 17 MPa. Following treatment, the compression stress ranged from 7 to 13.4 MPa, indicating that the exposure to a high pH solution facilitates the densification process. However, a reduction in flexural properties (MOR, LOP, and MOE) was observed on the alkali-treated samples after a three-point bending test. Physical properties (water absorption and thickness swelling) were not altered after delignification. These findings demonstrate that the direct application of a densification process optimized for natural bamboo is not fully effective for chemically modified bamboo, highlighting the need for adjustments. Delignified bamboo showed an increase in free space after chemical treatment, which should be further densified under higher degrees.

## 1. Introduction

Bamboo exhibits excellent mechanical properties, making it a promising alternative and sustainable material for construction applications. Despite this, a few factors hinder its use on a large scale, such as anisotropy in physical and mechanical properties along the culm, hygroscopicity, and susceptibility to degradation. These characteristics must be overcome to promote wider use for bamboo in the construction industry [1]. By modifying the physical and mechanical properties of bamboo through controlled processes, alternative products can be produced with greater standardization and reliability. Modifications such as chemical treatments and densification are alternatives that have been explored in the wood and bamboo industry to minimize the heterogeneity of these materials and increase their performance [2,3,4,5].

The densification process consists of compressing bamboo along its thickness in a thermo-hydraulic press system, resulting in the decrease in the internal volume of the material due to either the plasticization or collapse of cell walls [4,6,7]. To enhance wood quality, densification is employed in various processes such as wood shaping, molding, friction welding, embossing, and wood bending. These applications serve to dissipate internal stresses, improve key wood characteristics, including dimensional stability, strength, surface hardness, and durability, and facilitate the drying and plasticization of the wood. However, while processing in an open system, it is difficult to regulate the parameters such the moisture content of the raw materials. Whether the process is open or closed will affect the densification processing’s outcome [8].

The delignification process involves extracting the lignin from vegetal-based resources and disintegrating the lignocellulosic structure into fibrous components [9]. The delignification process can be carried out in two ways: the solvent process and the chemical process. The most used in industry is the chemical process using sulfite and alkaline pulping [9]. The delignification process using solvent-based pulping such as sodium hydroxide, anthraquinone, and methanol usually has higher efficiency, is cheaper, and presents lower emissions and by-products. Nonetheless, this process is mainly used on a small scale [9]. The use of water as a pretreatment to soften bamboo tissue has been studied, and it led to the appearance of less cracks during densification [10]. The two-step process of delignification/densification involves the partial removal of lignin and hemicellulose from the lignocellulose materials followed by thermo-mechanical pressing. This process has been used for wood or bamboo to achieve more efficient densification using less energy and slightly modifying their structure, resulting in better performance at biological resistance, dimensional stability, lower equilibrium moisture content, and improved durability [11]. However, most studies on bamboo densification have focused on untreated materials or closed-system pressing setups [4,7,11]. There is limited understanding of how open-system pressing parameters translate to chemically modified bamboo, particularly after alkali-based delignification, and whether these modifications significantly alter densification behavior and final properties.

A two-step process of delignification with NaOH + Na_2_SO_3_ solution, followed by densification, improved the strength of wood. For instance, densified wood with a lignin removal of 45% presented an ultimate tensile strength of 587 MPa in comparison to 51.6 MPa for natural wood, and 175.0 MPa for densified wood without delignification. The wood had its thickness reduced by 80% [11]. For *Phyllostachys bambusoides* bamboo species, an improvement in tensile (770 MPa) and flexural strength (327 MPa) was found after the delignification and densification processes in comparison to natural bamboo (298 MPa and 148 MPa for tensile and flexural strength, respectively). The bamboo had a densification degree of 70% [12]. In another study with wood, densification was carried out after delignification, and an increase in density of approximately 2.5 was achieved. Lignin loss due to chemical treatment varied between wood species from 21.1% to 39.7%. Nonetheless, a significant increase in compression resistance was observed in all directions [13]. Although expected, it is not clear whether a laterally closed system was used for the densification process to allow for higher densification degrees. In solid juniper wood, a closed pressing system was used to densify wood after kraft cooking delignification treatment [14]. The density increased from 2.1 to 2.5 times, the flexural strength increased by 70–90%, and the elastic modulus increased 6–7.2 times in comparison to undensified wood. It is worth mentioning that chemically treated wood had superior mechanical behavior in comparison to non-treated, dry, and densified wood.

For *Dendrocalamus asper* bamboo without delignification, the optimization of densification parameters was studied with the aim of preventing shear failure during pressing and improving dimensional stability. The highest density value (1.30 g/cm^3^) was achieved after 50% thickness reduction [7]. Nonetheless, chemically modified bamboo can present higher porosity and free space on its structure, as well as a reduction in fiber integrity and matrix cohesion.

Therefore, this study applies chemical modification and delignification using NaOH-based solutions at different temperatures combined with two types of drying processes prior to the densification of *D. asper* bamboo. The densification follows the parameters defined for natural bamboo, and the objective is to evaluate the transferability and limitations of these parameters when applied to chemically modified bamboo. To the best of the authors’ knowledge, this is the first study to apply a densification protocol optimized for untreated bamboo to alkali-delignified *Dendrocalamus asper* in an open pressing system.

This study seeks to answer the following question: Can optimized open-system densification parameters used for untreated bamboo be effectively applied to alkali-delignified *D. asper*, and how does chemical pretreatment influence mechanical, physical, and structural outcomes? The following sections detail the materials and methods used (Section 2), present and discuss the results (Section 3), and conclude with a summary of findings and recommendations for future research (Section 4).

## 2. Materials and Methods

### 2.1. Material and Sample Acquisition

Three-year-old mature *Dendrocalamus asper* Backer ex K. Heyne bamboo poles were harvested at an experimental field in the state of São Paulo, Brazil (21°59′ S 47°26′ W). The harvested culms with an average diameter of 18 cm were immersed in a solution of disodium octaborate tetrahydrate (DOT) to improve their durability [15]. The treated bamboo poles were stored in a protected environment at room temperature for four months until they reached an equilibrium moisture content of 9 to 11%. Samples with average dimensions of 100 mm in length and 30 mm in width were extracted from different internodes located in the middle part of the culm. The wall thickness of all samples varied between 13 and 14 mm prior to densification.

### 2.2. Methods

**Figure 1:**
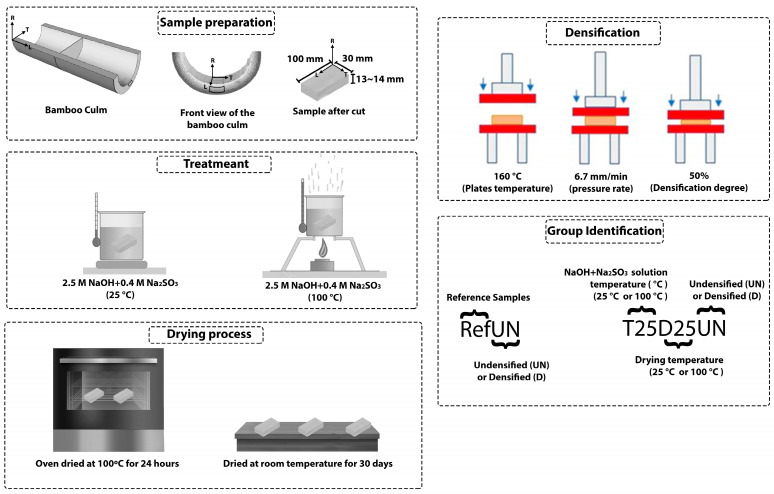
Sample preparation, treatment, and densification process and group identification.

**Figure 2:**
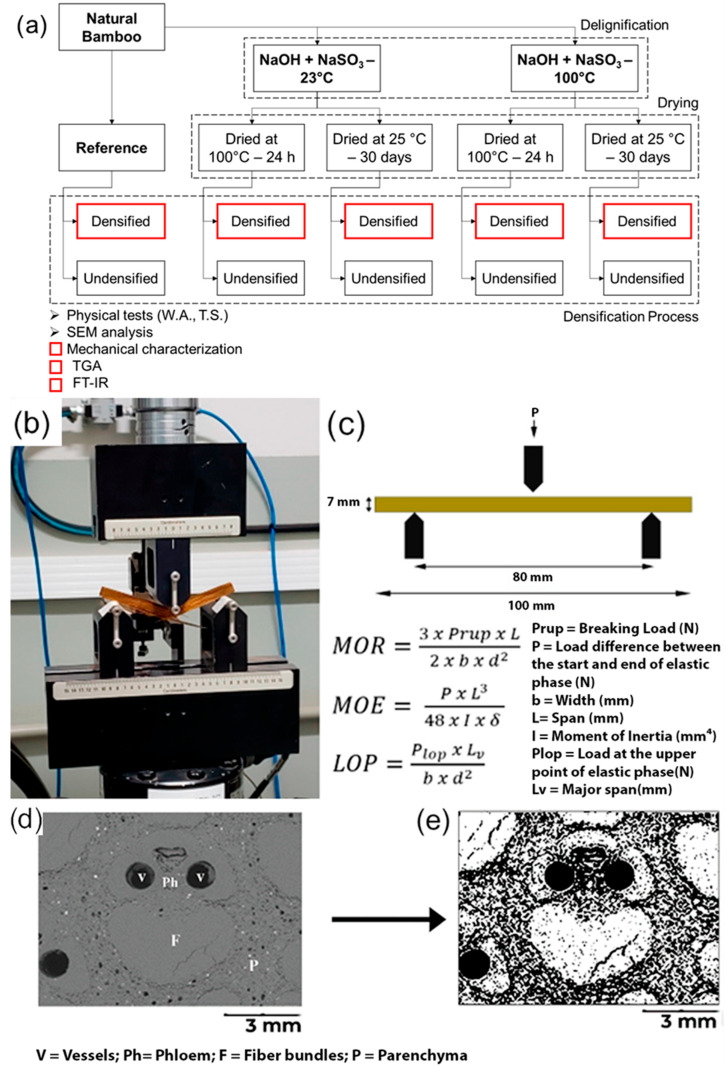
Flowchart of treatment and evaluation. (**a**) Bending test setup: (**b**) servo-hydraulic equipment, (**c**) three-point bending test scheme and formulations for mechanical properties, and Image J analysis: (**d**) reference SEM photo and (**e**) grayscale image with isolated fiber bundles.

The samples were submitted to delignification and a drying and densification process (Figure 1) and evaluated through physical, mechanical, chemical, and thermogravimetric tests. Before testing, the samples were conditioned at (25 ± 2) °C and 65% of relative humidity until reaching equilibrium with the environment. Each group received a code to facilitate its identification and analysis (Figure 1). The order of treatment and groups obtained are represented in Figure 2a. A summary of experimental procedures can be seen in Table 1.

#### 2.2.1. Delignification

The delignification process was carried out with a solution composed of sodium hydroxide 2.5 mol/L and Na_2_SO_3_ 0.4 mol/L. The bamboo samples were immersed in the alkali solution either at room temperature (25 °C) or 100 °C. To remove the solution after treatment, both processes were followed by immersion in boiling deionized water repeatedly until a neutral pH was achieved.

#### 2.2.2. Drying Process

Bamboo blocks obtained from each delignification process were submitted to two different drying processes. The first method was conditioning the samples in a controlled environment with 65% of relative humidity at room temperature (25 °C) for 30 days. The second method consisted of conditioning the samples in the oven at a temperature of 100 °C for 24 h.

#### 2.2.3. Densification Process

The samples were compressed radially in an open thermo-hydraulic press following the same process described by Kadivar et al. (2021) [7]. A universal testing machine EMIC 23–300 (INSTRON, São José dos Pinhais, PR, Brazil) with a load cell of 300 kN equipped with a small press plates system was used to apply a compression rate of 6.7 mm/min at 160 °C until the samples reached a densification degree of 50% of their thickness. After reaching the desired densification degree, the equipment was set to stop applying load and maintain the displacement. When the applied load was removed and the displacement is fixed, the internal stress began to decrease until it approached a constant value. The period required for internal stress to reach a constant value is called relaxation time. Data about the measured load were gathered for 10 min to analyze the relaxation behavior and obtain the relaxation curves.

#### 2.2.4. Physical Properties

Samples with dimensions of (21 × 21 × t) mm were prepared and dried in an oven for 24 h at (103 ± 2) °C. For density, water absorption, and thickness swelling, the dimensions and weight of bamboo samples were taken after oven drying and after immersion in water for 1 h and 24 h with a digital caliper (±0.01 mm precision) and a digital scale (±0.01 g precision). Density was calculated based on ASTM Standard D2395-17 recommendations [16]. Thickness swelling and water absorption values after 1 h and 24 h of water immersion were calculated in relation to the weight and dimensions of the samples after oven drying at (103 ± 2) °C. Statistical analyses were performed using RStudio version 4.2.0. A 95% confidence level was used for all comparisons. Data normality was assessed using the Shapiro–Wilk test prior to performing one-way ANOVA and Tukey’s HSD post-hoc tests. All the comparisons were made within the same condition (oven-dried, 1 h, or 24 h water immersion).

#### 2.2.5. Mechanical Analysis

Samples with dimensions of 7 mm thickness, 8 mm width, and 100 mm length extracted from the middle layer of specimens were prepared to perform three-point bending tests with a span of 80 mm following ASTM D790 test method recommendations [17]. A mechanical servo-hydraulic testing machine (MTS Model 370.02, Eden Prairie, MN, USA) was used to perform the tests (Figure 2b).

Modulus of rupture (MOR), modulus of elasticity (MOE), and limit of proportionality (LOP) were calculated based on the equations described in Figure 2c. Specific energy (EE) was calculated as the area under the stress–strain curve divided by the cross-section of the samples. One-way ANOVA and post-hoc Tukey tests were performed following a similar protocol to the previous subsection.

#### 2.2.6. Image Analysis

A Hitachi TM-3000 (Tokyo, Japan) Scanning Electron Microscope (SEM) using an accelerating voltage of 15 kV and a backscattered electron detector was used to obtain images from the cross-sectional area of the samples. To prepare the samples, the Struers Tegra Pol-11 machine (Champigny, France) was used for sanding and polishing. To achieve a highly reflective surface, samples were submitted to different grit size paper (from P320–P2000) with water, and polycrystalline diamond suspension solutions were used to finish the polishing process. ImageJ 1.53t software was used to calculate the fiber volume fraction of bamboo [18]. First, the images from the SEM were collected and the image was focused on the usable area. Secondly, the image was transformed into an 8-bit (grayscale) image to separate the fiber portion from the holes and parenchyma using the software command limit. The software automatically detects the darkest part, which is the parenchyma and the empty spaces. This process is exemplified in Figure 2d,e.

#### 2.2.7. Chemical Analysis

Fourier Transform Infrared Spectroscopy (FT-IR) analysis was performed on densified groups after delignification and drying process. Particles of bamboo were obtained from the analytical mill Quimis–Q298A, and a PerkinElmer Spectrum One spectrometer (Waltham, MA, USA) with ATR accessory was used to perform scans along the range of 4000 to 600 cm^−1^ with a resolution of 4 cm^−1^.

#### 2.2.8. Thermogravimetric Analysis

Natural and alkali-treated densified bamboo were used to perform simultaneous TG-DSC measurements, using a NETZSCH model STA 449 F3 apparatus (Selb, Germany), within a temperature range from 25 to 600 °C under a nitrogen environment, purged at 40 mL.min^−1^. A constant heating rate of 10 °C min^−1^ was maintained.

## 3. Results and Discussion

### 3.1. Physical Properties

**Figure 3:**
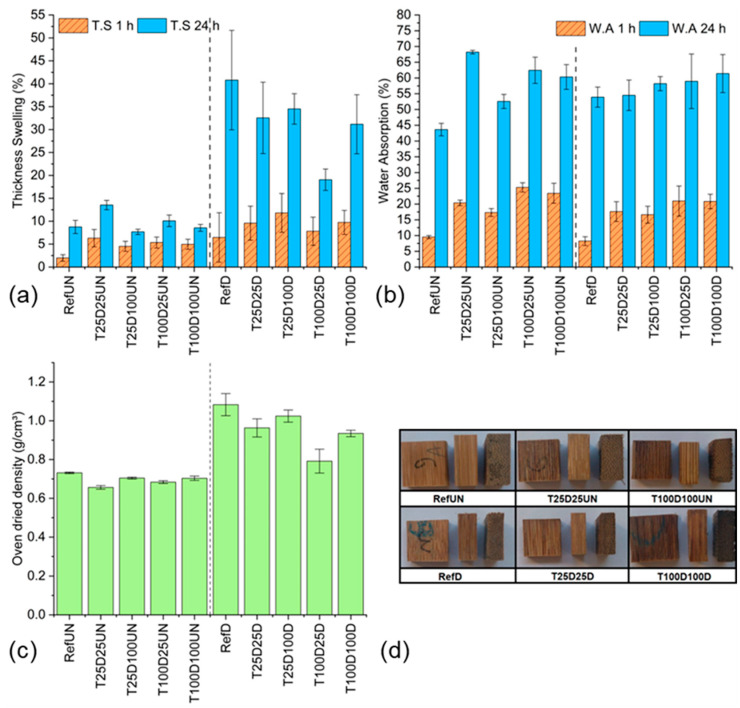
Bamboo oven-dried density values at different conditions (**a**). Bamboo water absorption values at different conditions (**b**). Thickness swelling for bamboo before and after treatment (**c**). Photos of bamboo samples before and after alkali treatment (**d**).

As shown in Figure 3a and detailed in Table 2, all alkali-treated groups exhibited lower oven-dried density after densification compared to the reference group (RefD: 1.083 g/cm^3^). The lowest value was found in group T100D25D (0.791 g/cm^3^), while T25D100D had the highest treated value (1.024 g/cm^3^). For non-densified samples, density ranged from 0.656 to 0.731 g/cm^3^, and no statistically significant differences were observed among them.

Regarding water absorption (Figure 3b and Table 3), after 24 h, this was significantly lower in the undensified reference sample (RefUN: 43.63%) compared to all chemically treated undensified samples (ranging from 52.56% to 68.21%). Among the densified groups, absorption ranged from 53.92% (RefD) to 61.40% (T100D100D), and no statistically significant differences were observed between treated and untreated densified samples after 24 h.

The thickness swelling results (Figure 3c and Table 4) reveal densified reference samples had the highest thickness swelling (40.79%), while alkali-treated densified samples—especially T100D25D—showed significantly reduced swelling (19.05%). Swelling values for undensified groups remained more stable and lower overall, ranging from 7.68% to 13.53%.

Figure 3d shows post-treatment photos of samples from all groups after 24 h water immersion. The images reveal clear signs of defibrillation and surface degradation in alkali-treated groups, particularly in densified samples.

The reduction in density after chemical treatment and densification can be attributed to the extraction of lignin and hemicellulose, which reduces the solid mass and creates more internal voids in the bamboo structure. This effect was most pronounced in T100D25D, indicating that exposure to higher temperature alkali treatment followed by slow drying compromises compactability in the open pressing system. This is further supported by a higher standard deviation in the density data, likely due to lateral material displacement, which is not constrained in an open press system.

Regarding water absorption, the increased uptake in chemically treated samples suggests that alkali delignification disrupted the microstructure, introducing gaps and micro voids. As noted by Yuan et al. [19], sclerenchyma tissue inherently absorbs more water than parenchyma, and the removal of lignin alone does not mitigate this effect. Densification typically reduces water uptake; but, in this case, the structural damage from chemical treatment appears to offset the benefit of compression.

**Figure 4:**
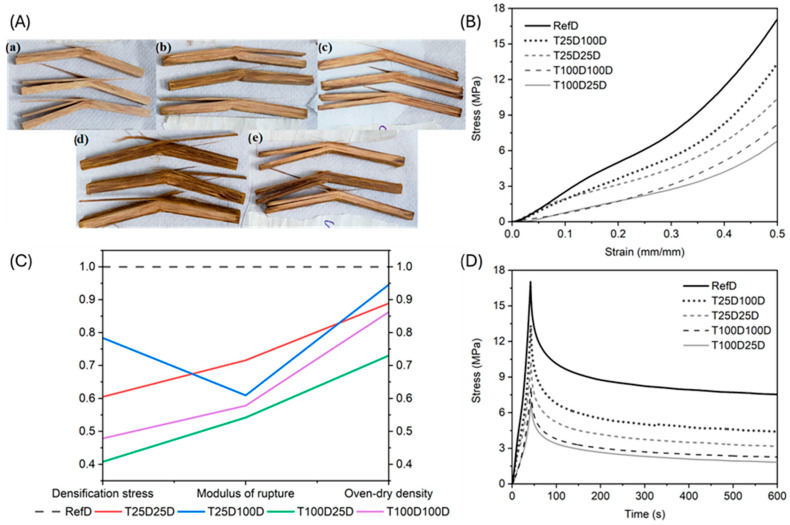
(**A**) Failure patterns: (**a**) RefD, (**b**) T25D25D, (**c**) T25D100D, (**d**) T100D25D, (**e**) T100D100D. (**B**) Characteristic stress–strain plot during the densification process. (**C**) Correlation between normalized densification stress and modulus of rupture and density. (**D**) Characteristic relaxation curve plot during densification process.

Despite its lower density, T100D25D demonstrated superior dimensional stability in terms of thickness swelling. This group’s behavior is linked to a higher porosity and stress relaxation capacity, as also seen in Figure 4D, where it reached the lowest residual internal stress (~2 MPa). The softened and more porous structure likely accommodated the thickness reduction more effectively without creating severe internal stresses.

While chemical treatment facilitated pressing by lowering compression stress, it also reduced structural integrity and densification effectiveness, as confirmed by physical properties, swelling trends, and the visual degradation seen in Figure 3d.

### 3.2. Mechanical Characterization

Mechanical performance after densification is summarized in Table 5. The reference group (RefD) exhibited the highest values across all measured properties: modulus of rupture (MOR) = 266.7 MPa, limit of proportionality (LOP) = 174.2 MPa, and modulus of elasticity (MOE) = 19.2 GPa. Among the chemically treated groups, the lowest MOR was observed in T100D25D (144.6 MPa), while T25D25D remained the highest MOR among them (190.9 MPa). MOE followed the same pattern, with T100D25D reaching a minimum of 12.9 GPa and T25D100D peaking at 15.8 GPa.

Specific energy (EE) values ranged from 13.0 to 20.4 kJ/m^2^ across all groups, with no statistically significant differences, as shown in Table 5. The LOP values showed similar reductions for treated groups, indicating earlier yield points under loading.

When normalized by density, the MOR of RefD (1.083 g/cm^3^) was approximately 246 MPa·cm^3^/g compared to 183 MPa·cm^3^/g for T100D25D (0.791 g/cm^3^). These normalized values partially close the performance gap, yet demonstrate that treated samples still suffer from reduced mechanical efficiency. Similarly, for LOP, the difference between reference and treated groups reduced, i.e., T100D25D was equivalent to 52% of the reference, and, after normalization, the percentage increased to 71%. Interestingly, for normalized MOE, the lowest value from treated groups (T100D25D) became the closest to the reference.

Figure 4A shows the failure patterns after bending tests. The untreated densified bamboo (RefD) primarily failed through longitudinal splintering from fiber rupture, while chemically treated groups exhibited a mixture of shear failure, inter-fiber delamination, and weaker bonding between vascular bundles and parenchyma. The middle and bottom samples in Figure 4A-a, especially from the T100D25D group, clearly show these effects. Notably, crack propagation in treated groups was slower, and boiling-treated samples (T100D25D and T100D100D) displayed more plastic deformation, suggesting that chemical treatment softened the matrix or decreased the stiffness due to having a lower density.

The load required to achieve 50% thickness reduction during densification is shown in Figure 4B through characteristic curves for each condition. Treated samples required less compressive stress (7–13.4 MPa) compared to the untreated RefD group (17 MPa), indicating easier compressibility after lignin and hemicellulose removal. The lowest densification stress (7 MPa) was required for T100D25D, the same group with the lowest final density (0.791 g/cm^3^) and lowest MOR (144.6 MPa). In contrast, RefD, which resisted densification the most, had the highest density and MOR, reinforcing the positive correlation between densification stress, final density, and mechanical performance.

Figure 4C visualizes this correlation, showing that, as densification stress increases, both density and MOR also increase. This suggests that compression resistance is a proxy for internal structure integrity; when structure is heavily weakened by chemical treatment, the bamboo compresses more easily, but yields inferior strength.

The characteristic internal stress relaxation behavior during densification is shown in Figure 4D. Across all groups, stress decayed rapidly during the first 50 s, followed by a slower decline toward an asymptotic residual value by 550 s. The residual internal stress for RefD was approximately 8 MPa, while the lowest was observed in T100D25D (~2 MPa). This further supports the fact that chemically treated bamboo experiences greater structural relaxation, which is consistent with both its softer mechanical response and its more plastic deformation behavior during mechanical testing.

The marked reduction in MOR and MOE in chemically treated bamboo confirms the fact that delignification degrades the mechanical integrity of the bamboo matrix, weakening the load transfer between fibers and parenchyma. Even though densification typically improves mechanical performance, in this case, chemical degradation outweighed the mechanical gains, especially for the more severe treatments like T100D25D.

Normalizing the MOR by density reduced the apparent performance gap between RefD and treated samples; yet, the treated groups still underperformed. This highlights the fact that the damage to the fiber–matrix interface and loss of structural stiffness cannot be fully recovered with densification alone, especially in an open pressing system where lateral restraint is absent.

As a performance benchmark, values from the literature for undensified *D. asper* bamboo indicate MOR and MOE of 215.3 MPa and 21.45 GPa, respectively [20]. This comparison emphasizes the fact that untreated bamboo can naturally outperform chemically treated and densified material if structural cohesion is lost during processing. Therefore, process control is crucial to ensure mechanical recovery.

**Figure 5:**
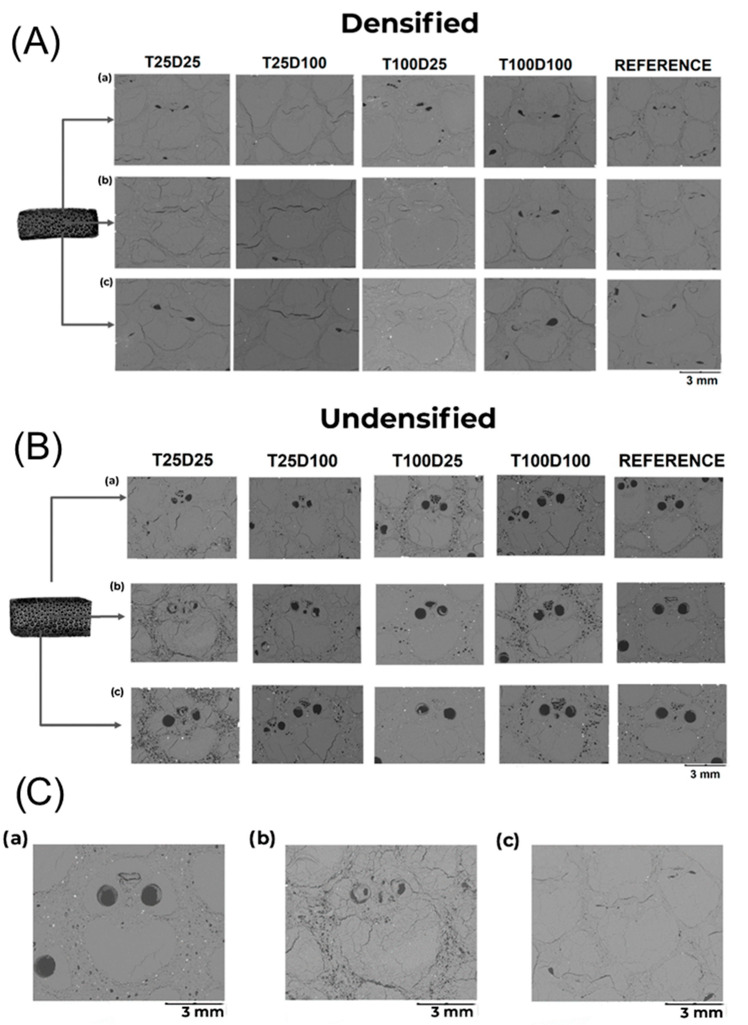
SEM images from densified bamboo samples. (**A**) The images were extracted from the (**a**) outer, (**b**) middle, and (**c**) inner layer. (**B**) SEM images from undensified bamboo samples: (**a**) outer, (**b**) middle, and (**c**) inner layer. (**C**) (**a**) Reference group without densification, (**b**) T25D25UN (alkali-treated non-densified), (**c**) densified reference group.

The similar specific energy across all groups indicates that energy absorption capacity was less affected, suggesting a retained deformation ability, even if strength decreased. However, failure behavior changed notably; treated groups were prone to fiber defibrillation, shear separation, and crack branching, as also observed in the SEM images (Figure 5C), which show fiber bundle detachment, vessel collapse, and weakened tissue connections.

Together, the data from Figure 4A–D and Table 5 show that mechanical performance, densification resistance, and final density are tightly linked, and that greater structural degradation leads to easier compression, lower strength, and lower internal stress retention. To counterbalance this effect, future studies should explore higher densification degrees, closed-system pressing, or less aggressive chemical treatments to achieve improved structural recovery and performance.

### 3.3. Image Analysis

Image analysis through ImageJ software (Table 6) showed that the average fiber fraction for bamboo increased after alkali treatment, with the highest fractions observed in room-temperature solutions. This is consistent with the removal of the lignin from the bamboo structure. However, for densified samples, treated groups presented lower values in comparison to the reference one. Notably, the volume of the fiber fraction of the T100D100D group was similar to that of the undensified reference, indicating an approximate reduction of 10%. This can be related to a reduction, to a lesser extent, in cellulose content, which becomes apparent after densification. It could also result from the limited area sampled in SEM analysis, which may not be representative of the entire cross-section, or, alternatively, it may indicate the need for a higher degree of densification.

Thermal analysis, which will be discussed in detail in the next section, indicates a loss of cellulose after treatment and densification. The images taken from the Hitachi TM-3000 Scanning Electron Microscope (SEM) showed characteristics of the densification processes and the influence of the alkaline solution (Figure 5A,B). Images were obtained from the inner, middle, and outer layers of the cross-section. It is possible to observe the vascular vessels on undensified groups (Figure 5C(a)) and the influence of the alkali treatment on them (Figure 5C(b)). Compared to the reference, a greater number of microcracks appeared in the bamboo structure of the treated samples due to lignin extraction and material degradation. Samples that underwent densification exhibited closed voids resulting from the process and showed fiber regions (lighter areas) positioned closer together (Figure 5C(c)). The use of boiling solution raised the number of visible cracks and space, allowing the material to better accommodate the displacement during the densification process. However, it reduces the mechanical properties of densified bamboo at the same densification degree, as the bond between matrix and fibers was weakened.

The reduction in fiber fraction in densified chemically treated bamboo may initially seem counterintuitive. However, it reflects the collapse of supporting parenchyma, the removal of binding components such as lignin and hemicellulose, and possibly even partial cellulose degradation, particularly at higher alkali concentrations and temperatures. While lignin removal facilitates the opening of the bamboo structure for densification, it simultaneously reduces tissue resilience and recoverability, resulting in less visible structural material per cross-section after compression.

The higher fiber fraction observed in room-temperature-treated undensified samples may result from greater selectivity in lignin removal, which preserves the fiber bundles while reducing amorphous content. However, this structural advantage is largely lost after pressing, unless the compression is high enough to restore compaction, which, in this study, using an open pressing system, was not fully achieved.

The SEM analysis confirms these trends. Cracks and voids formed during alkali treatment are visible in Figure 5C(b), especially in boiling-treated samples, which had the highest crack density. These cracks may facilitate compression by creating space for deformation, but they also weaken the fiber–matrix interface, as evidenced by the poorer mechanical results of the boiling-treated groups. In densified samples (Figure 5C(c)), the fiber bundles are closer together and voids are reduced, indicating effective compaction, but only when the matrix is not too degraded.

Thus, image analysis supports the mechanical findings: structure quality post-treatment is a balance between process severity and densification effectiveness. Over-treatment may improve compressibility but at the cost of inter-fiber bonding and mechanical recovery, reinforcing the need for optimized chemical exposure and pressing strategies.

### 3.4. Chemical and Thermogravimetric Analyses

**Figure 6:**
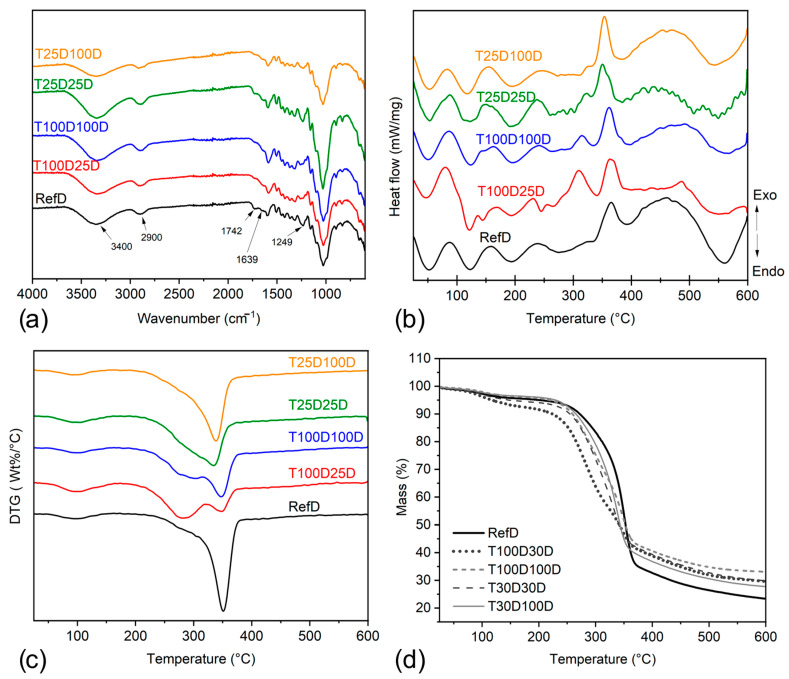
FT-IR spectra of densified bamboo samples (**a**), DSC curves of untreated and treated bamboo samples (**b**), DTG curves of untreated and treated bamboo samples (**c**), TG curves of untreated and treated bamboo samples (**d**).

On the FT-IR spectra, common lignocellulosic bands are identified (Figure 6a). The bands close to 1742 and 1249 cm^−1^ showed a decrease after the alkaline treatment. The band at 1742 cm^−1^ is attributed to C=O stretching vibration in hemicellulose in bamboo [21]. After treatment, this band is no longer observed in the samples, demonstrating the effectiveness of alkaline attack in removing hemicellulose from bamboo. Temperatures higher than 150 °C can also cause a removal of hemicellulose content [7,22]; however, the decrease in intensity was more visible in treated samples, which highlights the effect of the chemical treatment. The bands close to 1600 and 1505 cm^−1^ are attributed to the stretching of the benzene ring and -OCH_3_ groups in the lignin [23]. The intense absorption band close to 1049 cm^−1^ is associated with the C-O/C-C stretching vibrations [24,25]. The band close to 1249 cm^−1^ is attributed to the acetyl groups of lignin and presented a decrease in the intensity after the treatment with NaOH [26], which is related to the removal of lignin from the bamboo structure, with a greater decrease in treatments involving boiling alkali solution.

In regions close to 2900 cm^−1^, the band was associated with the symmetric and asymmetric stretching of CH_2_ groups. The band at 3400 cm^−1^ is related to the -OH stretching vibration from the cellulose of the bamboo fiber. The band of water adsorbed on the fibers is observed in the wavenumbers close to 1639 cm^−1^. In all the regions related to water absorption, there were no observed significant changes, which is in accordance with the results found on water absorption test after 24 h immersion for densified samples. These spectroscopic findings support the targeted removal of hemicellulose and lignin, particularly in group T100D25D, and partially explain the reduced matrix cohesion and altered failure patterns observed in SEM and mechanical tests.

Bamboo fibers are primarily composed of hemicellulose, cellulose, and lignin. Some other substances may be present as components or impurities on the surface of the fibers. The thermal decomposition of bamboo fibers can be divided into four main stages, i.e., moisture evaporation, hemicellulose decomposition, cellulose decomposition, and lignin decomposition [27]. The decomposition of hemicellulose occurs at lower temperatures, with a further decomposition of cellulose. Lignin is the component with the highest decomposition temperature and, according to some studies, represents the lowest rate of mass loss within the heating rate used [28]. Figure 6b shows the DSC curves of untreated and treated bamboo fibers. When temperatures are lower than 200 °C, it is possible to observe endothermic peaks related to the evaporation of moisture present on the surface of the fibers. Cellulose decomposition can be observed at temperatures close to 350 °C, with an endothermic peak appearing in all bamboo fiber samples. However, a decrease in peak intensity was noted in the treated groups dried at room temperature (T25D100D and T25D25D), which may be associated with an effective attack on bamboo cellulose by the alkaline chemical treatment. Two exothermic phenomena are observed at 275 °C and 365 °C, associated with the decomposition of hemicellulose and lignin, respectively. It is possible to observe a decrease in peak intensity at 365 °C for the T100D25 group. This result may be associated with better lignin removal when treating bamboo fiber with NaOH at 100 °C and drying at 25 °C [23,24,25,26,27,28,29]. Through the DTG curves of untreated and treated bamboo (Figure 6c), in the temperature range between 25 and 200 °C, a small loss of mass for all groups is observed, which was associated with the removal of moisture. A peak close to 300 °C is associated with the decomposition of hemicellulose and a peak close to 350 °C is related to the decomposition of cellulose in the fibers. It is evident that samples without alkaline treatment exhibited a more intense peak between 300 °C and 350 °C than those treated with NaOH. This thermal degradation pattern is consistent with the microstructural damage observed in SEM images (Figure 5C), particularly the increased void formation and fiber–matrix separation in chemically treated groups. The weakened bonding at the interface between parenchyma and sclerenchyma contributes to the observed reduction in mechanical strength after densification (Table 5). The reduction in the cellulose content of treated samples corroborates with the loss of mechanical resistance after pretreatment with the same densification parameters, indicating that the alkali treatment caused the expected removal of lignin content, but it also led to a degradation on the interface between parenchyma and sclerenchyma and the removal of cellulose content. Figure 6d shows the TG curves of untreated and treated bamboo samples. From the TG curves, the mass loss before and after the treatments are very similar, and it is possible to observe a greater mass loss for the untreated fiber. This result may be associated with the presence of alkalis on the surface of the treated samples that do not evaporate within the studied temperature range. When these results are considered alongside fiber volume fraction data (Table 6), they suggest that the effective thermally stable mass per unit of structural material was reduced in treated groups. Despite this, specific energy values (EE) remained relatively unchanged (Table 5), indicating that, while the structural strength decreased, the ability of the material to absorb energy under load was preserved.

Chemical and thermal analysis confirms that alkaline treatment effectively removes hemicellulose and lignin but also partially degrades cellulose, especially in high-temperature or prolonged treatments. These changes reduce both thermal stability and structural cohesion, leading to weakened fiber–matrix bonding and impaired mechanical recovery. The results demonstrate that, while delignification facilitates densification by lowering resistance, it compromises performance, particularly in open pressing systems where lateral confinement is absent. Exploring higher densification degrees on closed-system pressing may be necessary to fully recover mechanical function in delignified bamboo.

## 4. Conclusions

This study evaluated whether a thermo-mechanical densification protocol developed for untreated *Dendrocalamus asper* bamboo could be directly applied to alkali-delignified material using an open pressing system. The results showed that, while chemical pretreatment significantly reduced compressive resistance and facilitated densification, it also led to microstructural degradation and compromised mechanical recovery. Specifically, the delignified bamboo exhibited lower density after pressing, reduced stiffness and strength, and increased internal porosity, indicating that the original densification parameters are not fully transferable to chemically modified bamboo without adjustment.

These findings carry broader implications for sustainable material engineering, particularly in the development of renewable high-performance bamboo composites for structural applications. Although alkali treatments improve processability, they also alter the fiber–matrix interface and reduce the capacity to regain strength through compression alone.

Future work should investigate closed-system pressing with optimized densification degrees to better control compaction in modified bamboo. This study was limited by a single pressing setup, which limited higher densification degrees, and did not examine long-term performance. Further research is needed to evaluate the moisture sensitivity, durability, and scalability of treated bamboo materials under service-like conditions. Life cycle assessment would be beneficial to understand the impact of chemical treatment on the environmental footprint of the process and product.

## Figures and Tables

**Table 1:** Experimental procedures for evaluating *D. asper* bamboo after alkali treatment.

Type of Test	Dimensions (L × W × T *) (mm)	Number of Samples Per Group	Total Number of Samples	Treatment
Physical properties	21 × 21 × (6.5–7)	5	25	Densified
	21 × 21 × (13–14)	3	15	Undensified
Image analysis	21 × 21 × (6.5–7)	1	5	Densified
	21 × 21 × (13–14)	1	5	Undensified
Three-point bending test	100 × 8 × 7	9	45	Densified
TGA andFT-IR	Bamboo powder	-	-	Densified

* L: length, W: width, T: thickness.

**Table 2:** Density values for bamboo with statistical comparison.

Density (g/cm^3^)—Mean (SD)			
Undensified groups			
Treatment	Oven-dried	1 h water immersion	24 h water immersion
RefUN	0.731 ^a^ (0.004)	0.773 ^a^ (0.021)	0.907 ^a^ (0.005)
T25D25UN	0.656 ^a^ (0.010)	0.714 ^a^ (0.008)	0.897 ^a^ (0.012)
T25D100UN	0.704 ^a^ (0.005)	0.747 ^a^ (0.020)	0.945 ^a^ (0.014)
T100D25UN	0.683 ^a^ (0.007)	0.792 ^a^ (0.0007)	0.946 ^a^ (0.024)
T100D100UN	0.703 ^a^ (0.011)	0.788 ^a^ (0.022)	0.969 ^a^ (0.015)
Densified groups			
Treatment	Oven-dried	1 h water immersion	24 h water immersion
RefD	1.083 ^a^ (0.057)	1.078 ^a^ (0.022)	1.076 ^a^ (0.042)
T25D25D	0.963 ^b,c^ (0.047)	1.009 ^a^ (0.039)	1.067 ^a^ (0.022)
T25D100D	1.024 ^a,c^ (0.031)	1.039 ^a^ (0.049)	1.289 ^a^ (0.421)
T100D25D	0.791 ^d^ (0.062)	0.740 ^b,c^ (0.333)	1.017 ^a^ (0.055)
T100D100D	0.934 ^b^ (0.017)	1.001 ^a,c^ (0.013)	1.083 ^a^ (0.026)

^a,b,c^ letters indicate significant statistical differences between values in the same column (Tukey).

**Table 3:** Bamboo water absorption with statistical comparison.

Water Abs (%)—Mean (SD)		
Undensified groups		
Treatment	1 h	24 h
RefUN	9.51 ^a^ (0.50)	43.63 ^a^ (2.00)
T25D25UN	20.36 ^b,c^ (0.88)	68.21 ^b^ (0.59)
T25D100UN	17.29 ^b^ (1.25)	52.56 ^a,c^ (2.22)
T100D25UN	25.27 ^c^ (1.48)	62.46 ^b,c^ (4.16)
T100D100UN	23.40 ^b,c^ (3.18)	60.32 ^b,c^ (3.94)
Densified groups		
Treatment	1 h	24 h
RefD	8.23 ^a^ (1.41)	53.92 ^a^ (3.20)
T25D25D	17.59 ^b^ (3.16)	54.51 ^a^ (4.84)
T25D100D	16.59 ^b^ (2.69)	58.21 ^a^ (2.25)
T100D25D	20.93 ^b^ (4.79)	58.95 ^a^ (8.65)
T100D100D	20.80 ^b^ (2.29)	61.40 ^a^ (6.03)

^a,b,c^ letters indicate significant statistical differences between values in the same column (Tukey).

**Table 4:** Thickness swelling for bamboo with statistical comparison.

Thickness Swelling (%)—Mean (SD)		
Undensified groups		
Treatment	1 h	24 h
RefUN	1.98 ^a^ (0.71)	8.75 ^a^ (1.43)
T25D25UN	6.29 ^a^ (1.90)	13.53 ^a^ (1.02)
T25D100UN	4.52 ^a^ (1.09)	7.68 ^a^ (0.58)
T100D25UN	5.34 ^a^ (1.21)	10.08 ^a^ (1.27)
T100D100UN	4.97 ^a^ (1.10)	8.55 ^a^ (0.76)
Densified groups		
Treatment	1 h	24 h
RefD	6.46 ^a^ (5.39)	40.79 ^a^ (10.85)
T25D25D	9.56 ^a^ (3.72)	32.55 ^a^ (7.79)
T25D100D	11.81 ^a^ (4.24)	34.50 ^a^ (3.33)
T100D25D	7.81 ^a^ (3.08)	19.05 ^b,c^ (2.33)
T100D100D	9.74 ^a^ (2.63)	31.15 ^a,c^ (6.44)

^a,b,c^ letters indicate significant statistical differences between values in the same column (Tukey).

**Table 5:** Mechanical properties of *D. asper* bamboo in different conditions.

Treatment	MOR (MPa)	S-MOR (MPa)	LOP (MPa)	S-LOP (MPa)	MOE (GPa)	S-MOE (GPa)	EE (kJ/m^2^)
RefD	266.7 ^a^ (27.8)	246.3	174.2 ^a^ (23.5)	160.8	19.2 ^a^ (2.6)	17.7	19.5 ^a^ (10.5)
T25D25D	190.9 ^b^ (13.9)	198.2	118.3 ^b^ (9.71)	122.8	14.7 ^b^ (1.3)	15.3	20.4 ^a^ (3.3)
T25D100D	162.5 ^c^ (19.2)	158.7	118.7 ^b^ (17.8)	115.9	15.8 ^b^ (2.6)	15.4	20.2 ^a^ (5.9)
T100D25D	144.6 ^c^ (18.3)	182.8	90.9 ^c^ (10.9)	114.9	12.9 ^b^ (3.2)	16.3	14.1 ^a^ (6.9)
T100D100D	154.2 ^c^ (20.0)	165.1	102.1 ^b,c^ (23.2)	109.3	14.6 ^b^ (2.3)	15.6	13.0 ^a^ (0.8)

^a,b,c^ letters indicate significant statistical differences between values in the same column (Tukey). S stands for specific property, which is normalized by oven-dried density.

**Table 6:** Values of average fiber fraction for each bamboo group.

Treatment	Average Fiber Fraction
RefUN	52.13%
T25D25UN	65.98%
T25D100UN	63.29%
T100D25UN	60.92%
T100D100UN	59.71%
RefD	66.15%
T25D25D	61.64%
T25D100D	62.45%
T100D25D	54.84%
T100D100D	52.81%

## Data Availability

Data will be made available upon request.
